# The Global Threat of Counterfeit Drugs: Why Industry and Governments Must Communicate the Dangers

**DOI:** 10.1371/journal.pmed.0020100

**Published:** 2005-03-14

**Authors:** Robert Cockburn, Paul N Newton, E. Kyeremateng Agyarko, Dora Akunyili, Nicholas J White

## Abstract

The production of substandard and fake drugs is a vast and underreported problem, particularly affecting poorer countries. Cockburn and colleagues argue that the pharmaceutical industry and governments must both take action

## Introduction

The production of substandard and fake drugs is a vast and underreported problem, particularly affecting poorer countries. It is an important cause of unnecessary morbidity, mortality, and loss of public confidence in medicines and health structures. The prevalence of counterfeit drugs appears to be rising (see “The Scale of the Problem”) and has not been opposed by close cooperation between drug companies, governments, or international organizations concerned with trade, health, customs and excise, and counterfeiting.

In this article we suggest that many pharmaceutical companies and governments are reluctant to publicize the problem to health staff and the public, apparently motivated by the belief that the publicity will harm the sales of brand-name products in a fiercely competitive business. Publicly, at least, several industry sources say the justification for secrecy is to avoid any alarm that could prevent patients taking their genuine medicines. We argue that this secrecy, and the subsequent lack of public health warnings, is harming patients and that it is also not in the long-term interests of the legitimate pharmaceutical industry. We urge a change to mandatory reporting to governmental authorities, which should also have a legal duty to investigate, issue appropriate public warnings, and share information across borders. This is not a role for the pharmaceutical industry, which has a serious conflict of interest.

While some drug companies have given public warnings to protect patients, others have been criticized for withholding information and, in a recent development in the United States, taken to court for failing to act. The industry is addressing the problem. In 2003, US pharmaceutical companies made an agreement with the US Food and Drug Administration (FDA) that they would report suspected counterfeit drugs to the FDA within five days of discovery (see “Companies That Have Warned”), although this remains a voluntary arrangement. In many poorer countries, where the problem is at its worst, there are no similar governmental and industry initiatives.

## The Scale of the Problem

It has been estimated that up to 15% of all sold drugs are fake, and in parts of Africa and Asia this figure exceeds 50% ([1,2,3,4,5,6,7]; R. Jones, FDA spokesperson, E-mail statement, 18 November 2004). The FDA estimates that fake drugs comprise approximately 10% of the global medicine market (R. Jones, FDA spokesperson, E-mail statement, 18 November 2004). This estimate suggests annual criminal sales in excess of US$35,000,000,000 [1,2]. The number of investigations of possible counterfeit drugs by the FDA has jumped from about five per year in the 1990s to more than 20 per year since 2000 (Figure 1).

**Figure 1:**
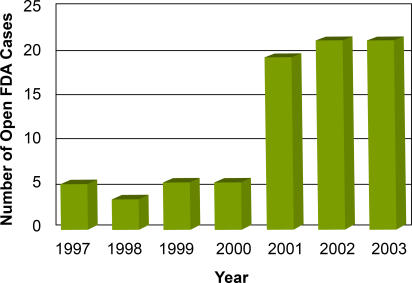
The Number of Investigations of Possible Counterfeit Drugs by the FDA Has Been Rising (Figure: Margaret Shear, Public Library of Science, adapted from [39])

Most of the literature on fake drugs derives from local investigative journalism [6,8,9,10,11,12,13,14], with little scientific public health enquiry relative to the enormous scale of this criminal enterprise. The effects on patients of counterfeit medicines are difficult to detect and quantify, and are mostly hidden in public health statistics. The estimate of 192,000 patients killed by fake drugs in China in 2001 gives an indication of the scale of human suffering (see Sidebar).

## Secrecy and Counterfeit Medicines

Most data on the epidemiology of counterfeit drugs are kept secret by the pharmaceutical industry and by governmental agencies. Drug companies employ investigators to track down and facilitate the shutting down of counterfeit industries, but this occurs very much in private.

There are no reliable accessible databases whereby health workers or the public can access current details of which products are being faked in a locality. It is obviously correct that information on anti-counterfeiting strategies and the sources of undercover intelligence should not be released, but we believe that the information on what drug is being counterfeited, and where, should be public knowledge [1].

## Government Reluctance

Governments are also often reluctant to publicize problems with the quality of the drug supply in their country. This is reflected in the lack of action in much of the world regarding the problem of counterfeits, relative to their large impact on public health. The World Health Organization (WHO) has a reporting system and some of the information is publicly available [15]. The public information, crucially, does not include the country or region where the fakes were identified. However, the WHO has received no reports of counterfeit drugs from member countries after 2002, and it received only 84 reports between 1999 and 2002 [16,17].

In some countries, government officials have been accused of involvement in the false certification of counterfeit drugs, while in others, governmental agencies have been criticized for suppressing information [9,18]. The WHO in the Western Pacific region, an area severely affected by counterfeit drugs, is planning a rapid alert system for expediting the sharing of warnings and information between governments in the region.

## Pharmaceutical Industry Survey

We wrote to the Pharmaceutical Security Institute (PSI) (see Box 1), which collates information on fake drugs obtained by the industry, asking whether they currently forwarded reports of counterfeit drugs to the relevant governments and the WHO. This question was not answered, but the PSI (in a letter dated 29 July 2003) informed us that, “Since its inception, it was recognized that a great deal of this information it [the PSI] contains would remain confidential and would not be disseminated. There is proprietary information that cannot be disclosed, either to peer member companies or to the general audience. Consequently, at this time the dissemination of information…is restricted and limited.” The letter added that the PSI encourages its members to report counterfeiting incidents to the appropriate authorities, and that it fully supports the voluntary reporting to the FDA. We also wrote to 21 major companies, of the more than 70 pharmaceutical companies with offices in the United Kingdom, asking for information on the companies' policies on what action should be taken and who should be told when one of their products was found to be counterfeited. We have received replies from six companies; one (Merck Sharp and Dohme) declined to give any information, while three (GlaxoSmithKline [GSK], Bristol-Myers Squibb, and Novartis) stated that they would inform the local drug regulatory authority if they were notified that one of their products was being counterfeited.

**Box 1. The Pharmaceutical Security Institute**

The PSI is a not-for-profit corporation formed by the major drug companies to collate their fake drug information to cooperate in fighting the racket. Based in Vienna, Virginia, United States, the PSI holds the only known comprehensive and updated source of fake drug information. The PSI Web site (www.psi-inc.org) states, “On a daily basis, many individuals unknowingly risk death or serious injury to their health by taking counterfeit pharmaceuticals.” But its databank, which health workers see as holding key information to prevent patients from taking life-threatening fakes, is not accessible to the WHO, health authorities, or the public. Such is the secrecy of the PSI's information, that access is restricted even between its member companies, which include the 15 largest drug manufacturers.

**Figure 2:**
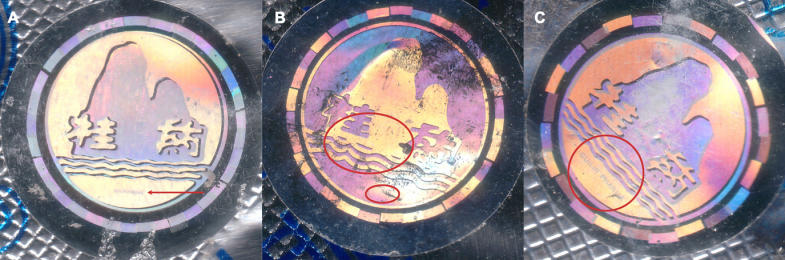
Genuine and Fake Guilin Pharma Artesunate Blister Pack Holograms Found in Mainland Southeast Asia (A) is the genuine hologram attached to the blister packs of the genuine Guilin Pharma artesunate. The red arrow points to a legend stating “GUILIN PHARMA”, which is visible with the naked eye as a thin strip below the waves, but can only be read with a microscope (letters are about 0.1 mm high). (B) is a fake artesunate blister pack hologram: the upper red ring shows that the hologram has crescents, rather than a continuous blank line, between mountain and waves, and the lower ring shows that there is no “GUILIN PHARMA” legend. (C) is also a fake artesunate blister pack hologram: the red ring shows that the “GUILIN PHARMA” legend is present but the letters are of larger font than those on the genuine hologram and can be read with the naked eye (letters are about 0.3 mm high). A warning sheet giving more details and photographs is available in [47]. (Photos: Paul Newton, Wellcome Trust SE Asian Tropical Medicine Research Units)

## Paucity of Warnings about Fake Drugs

That many pharmaceutical companies, professional organizations, and governments, both in developed and developing countries are not releasing warnings is manifested by the paucity of warnings relative to the scale of the problem. The industry's history of secrecy over data about fake drugs, and claims of a commercial motivation, go back over 20 years. In 1982, a spokesperson for the Association of the British Pharmaceutical Industry said, “It is difficult to declare a [fake drug] problem without damaging legitimate business” [13]. This impression of secrecy is supported by historical statements, such as the following: “The Society [Royal Pharmaceutical Society of Great Britain] is not issuing press releases [about counterfeit drugs] because it believes that as much as possible should be done behind the scenes…and that no great publicity should be sought because it could damage public confidence in medicines” [19]. But the Royal Pharmaceutical Society of Great Britain has recently revised its position. David Pruce, Director of Practice and Quality Improvement for the organization, told us (E-mail letter, 14 February 2005), “If there is a risk that a patient has been dispensed a counterfeit medicine, then it is vital that they are informed. There have been two recent cases in Great Britain where counterfeit medicines appeared in the legitimate pharmacy supply chain. The public announcement of the problem of the counterfeit medicines was therefore entirely proper and necessary.” He added, “It is important that news stories of this type are handled responsibly so that the public's confidence in their medicines is not undermined. This could deter patients from taking genuine medicines.”

**Recent Examples of Counterfeit Drugs**

- Approximately one-third to one-half of packets of artesunate tablets, the pivotal, life-saving anti-malarial drug, recently bought in Southeast Asia were fakes, containing no active ingredient at all. A nongovernmental organization in a Southeast Asian country bought 100,000 inexpensive “artesunate” tablets only to find that they were counterfeit [7,39]. See Figure 2 for examples of fake artesunate being sold in mainland Southeast Asia.
- A total of 192,000 Chinese patients are reported to have died in 2001 from fake drugs, and in the same year Chinese authorities “closed 1,300 factories while investigating 480,000 cases of counterfeit drugs worth 57 million USD” [12]. In 2004, Chinese authorities arrested 22 manufacturers of grossly substandard infant milk powder and closed three factories after the death of over 50 infants [40].
- In North America, counterfeit atorvastatin [41], erythropoietin [41], growth hormone [33], filgrastim [33,41], gemcitabine [36,37], and paclitaxel [36,37] have been reported recently.
- Nigeria recently threatened to ban the import of all drugs from India, a major supplier, because of the high prevalence of counterfeits amongst the imports [42].
- In Haiti, Nigeria, Bangladesh, India, and Argentina, more than 500 patients, predominantly children, are known to have died from the use of the toxin diethylene glycol in the manufacture of fake paracetamol syrup [43,44,45].
- During the 1995 meningitis epidemic in Niger, the authorities received a donation of 88,000 Pasteur Merieux and SmithKline Beecham vaccines from neighbouring Nigeria. The drugs were found to be counterfeit, with no traces of active product. Some 60,000 people were inoculated with the fake vaccines [24].
- The recent discovery of counterfeit antiretrovirals (stavudine-lamivudine-nevirapine and lamivudine-zidovudine) in central Africa [46] raises the prospect of a disastrous setback in the treatment of AIDS in sub-Saharan Africa, unless vigorous action is taken now.

This assessment, that the dangers of causing alarm amongst the general public could outweigh the benefits of disclosure, remains widespread in public statements. A spokesperson for the Association of British Pharmaceutical Industries, Marjorie Syddall, wrote (E-mail letter, 20 October 2003), “A company should be completely satisfied that a medicine is counterfeit before informing the authorities, but more importantly still, before it makes this information known to the public—so that no unnecessary alarm is caused.”

## Commercial Motivation—“Cut-Throat Competition”

Chris Jenkins, a founding member of the PSI, now Associate Director of Pinkerton Consulting and Investigations, told us (E-mail statement, 9 December 2004), “It is necessary to keep fake drug information confidential for commercial reasons…to avoid media leaks and to prevent the possibility of rival drug companies taking unfair commercial advantage of a victim company.” He explained, “At the outset, we [the PSI] were against having data online that anyone could interrogate…If a patient came to harm as a result of a counterfeit product, the company's good reputation is in danger of disappearing, together with a loss of confidence in the products… The one thing we were trying very hard to do was to keep it [data] out of the hands of the commercial people in any of the companies…The importance of meeting sales' targets is such that you can even find cut-throat competition between different operating divisions of the same company, let alone between two companies competing in the same market with similar drugs.”

The WHO 1999 guidelines for the development of measures to combat counterfeit drugs states that “the reluctance of the pharmaceutical industry, wholesalers and retailers to report drug counterfeiting to the national drug regulatory authorities could impede the national authorities from successfully taking measures against counterfeiting”, and suggests “the compulsory reporting to the relevant authorities of any incidents in which counterfeits are detected or involved” [20]. A recent review of the law and counterfeit drugs calls for the “eradication of the clandestine status of records and counterfeit drug information” [21]. At the International Conference of Drug Regulatory Authorities in Madrid in February 2004, it was stated by the WHO that “the drugs industry had a great deal of data but was ‘very reluctant to make them available’” [17].

## Information Strictly Confidential

In the US it was reported that it had been “very difficult to obtain citable factual information about the extent of the problem of counterfeit drugs. Drug companies keep the information they have strictly confidential” [22]. In 1989, the British Department of Health and Glaxo (now a part of GlaxoSmithKline) were criticized for not publicizing information about the discovery in Britain of fake Glaxo Ventolin asthma inhalers. London's *The Times* obtained the fake Ventolin's licence and batch numbers for a story, prompting the release of the information. Warning letters, drafted by Glaxo and the Department of Health, were sent to all 14,000 pharmacists in Britain five weeks after the fake's discovery [8]. In 1998, the company Schering do Brasil was accused of keeping secret the discovery of oral contraceptive pills made of wheat flour for 30 days while they carried out their own investigation [23]. According to the *Far Eastern Economic Review*, the company was fined US$2.5 million by the Brazilian government [6]. Schering do Brasil informed us (E-mail letter, 17 February 2005) that “Federal Justice cancelled the fine in 2002 after the company appealed”. In Niger, in 1995, one of the fake meningitis vaccines originating from Nigeria was labelled as made by SmithKline Beecham, but *Le Monde* reported that the company did not act against the counterfeiters, afraid that it might damage trade [24].

## Fake Paediatric Anti-Malarial Drugs

The need to release fake drug information is acute in Africa, where a resurgence of malaria is killing an estimated one million people a year, the vast majority of them children under five [25]. One example highlights the problems encountered. One of us (K. Agyarko) found counterfeits of the GSK paediatric anti-malarial syrup halofantrine (Halfan) in August 2002 in Ghana. That month he prepared a public health warning. Agyarko and his deputy told the BBC [26] that he also alerted GSK's Ghana agent, who visited him with staff from GSK's London headquarters and took away samples of the fake Halfan. Agyarko publicly stated (on 23 September 2002, at the First Global Forum on Pharmaceutical Anticounterfeiting in Geneva, Switzerland) [26] that he was asked by GSK to withhold his public warning because it would “damage” their product. After his meeting with GSK, no warning was issued. In a written statement (E-mail letter, 24 October 2003), GSK denied receiving Agyarko's fake Halfan alert and said the company was “not provided with any samples of fakes by the authorities in Ghana”.

After a year of enquiries, resulting in a BBC Radio programme (BBC Radio 4, “File on 4”, 5 October 2004) [26], GSK reversed its position and said that its local agent had “bumped into” Agyarko and had received his alert and samples of fake Halfan syrup. In a new statement (E-mail letter, 5 October 2004) GSK said: “At no point was any pressure put on the Ghanaian authorities not to issue a public warning on fake Halfan.” GSK's vice president of communications, Louise A. Dunn, told us (E-mail letter, 6 October 2004), “There was some confusion over the interactions with Mr Agyarko. The key point here is that there was no wrong doing…”

However, the Ghana incident needs to be viewed in the context of the wider illegal trade in fake Halfan syrup identified in West Africa, and GSK's reluctance to give us details about this trade. We asked GSK whether it had issued any public warnings about fake Halfan syrup, but the question was not answered. The only reference to counterfeit halofantrine syrup that we have been able to find in the public domain was published in a specialist technical journal that described the mass spectroscopy analysis of fake halofantrine syrups by the GSK Medicines Research Centre [27] and demonstrated that the fake syrups contained two potentially harmful sulphonamide drugs, but no halofantrine. We wrote to GSK (letter, 20 June 2003) asking when and where discoveries of fake Halfan were made, and whom GSK had informed about them. GSK told us only that “counterfeit Halfan is present in Nigeria and Sierra Leone” (letter, 21 July 2003). It gave no details of preparation type or discovery dates.

Fake GSK Halfan syrup was discovered in Nigeria in June 2002 by the Nigerian National Agency for Food and Drug Administration and Control. NAFDAC alerted GSK and issued a public health warning in June 2002 in the regular NAFDAC fake drug bulletin [28], giving the fake Halfan syrup's identifying details. The NAFDAC's Dora Akunyili told BBC Radio (5 October 2004): “It is more dangerous not to alert the public. We will still issue a warning even if we find it in only one shop. If you find any fake drug product in only one shop you can be sure it is in many villages…We don't defend companies. We are defending the people” [26].[Fig pmed-0020100-g004]

**Figure 3 pmed-0020100-g004:**
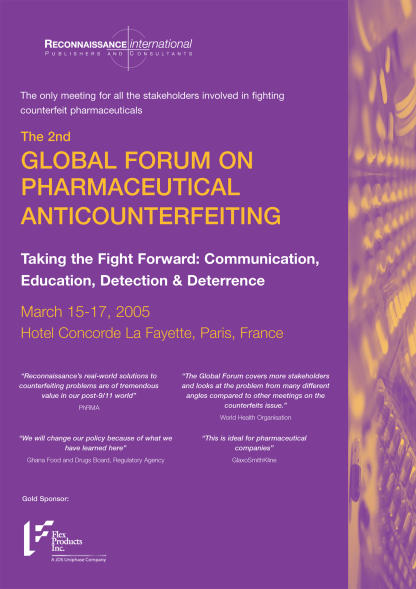
Poster Advertising the Second Global Forum on Pharmaceutical Counterfeiting (Figure: Ian Lancaster, Reconnaissance International)

The Pharmaceutical Board of Sierra Leone, which handles fake drug cases, was not informed by GSK of any discoveries of fake GSK Halfan syrup, according to its director Michael J. Lansana (E-mail letter, 21 January 2004), although it did receive a report of counterfeit adult Halfan caplets from GSK. Later, GSK told us (E-mail letter, 6 October 2004) the fake Halfan syrup it had tested was found in Sierra Leone in late 2001, and that it had informed Sierra Leone's Minister of Health and Sanitation of the find.

Only a single report of counterfeit halofantrine, which does not specify details of preparation type or location, is given in the WHO Counterfeit Drug Reports for 1999–October 2000 [15].

## Cross-Border Threats and Cooperation

The fake Halfan syrup cases highlight the importance of communication and cross-border cooperation, and the need for industry and governments to inform neighbouring countries when a fake is found. The global distribution and the scale of the racket in fake adult Halfan capsules was clear in December 2000, when Belgian customs seized 57,600 packs of fake GSK Halfan capsules (and 4,400 packs of fake GSK Ampiclox [ampicillin] and 11,000 packs of fake GSK Amoxil [amoxicillin]) en route from China to Nigeria. The counterfeiters in China were found to be preparing to export 43 tons of 17 brands of drugs from seven international pharmaceutical companies [29].

## Companies That Have Warned

Sometimes pharmaceutical companies have publicized information to alert health workers and patients and governments to the dangers of counterfeited or tampered products. For example, Johnson and Johnson, Serono, Hoechst, Wellcome Foundation (now part of GSK), GSK, and Genentech have publicized information on their drugs that have been counterfeited or tampered with. In 1982, cyanide-laced paracetamol killed seven people in the US. The pharmaceutical company whose product had been tampered with, Johnson and Johnson, issued alerts and cooperated with the investigation, and although the financial cost to the company was large, its long-term reputation was probably enhanced. Other companies, at least initially, did not take advantage of the disaster for their own financial gain [30]. In 2002, Johnson and Johnson issued 200,000 letters to health-care professionals in the US warning them of fake Procrit (erythropoetin) within one week of being notified of a severe counterfeit problem [31]. In 1982, Hoechst voluntarily took out magazine adverts in Lebanon to warn pharmacists and customers of a fake of its drug Daonil (glibenclamide) for the treatment of diabetes mellitus [13]. In 2001, Serono was told by the FDA to issue a public warning to hospitals, clinics, and patients in seven US states after the discovery of a counterfeit of its drug Serostim, a human growth hormone used in the treatment of AIDS and other conditions [32]. In 1984, in Thailand, the Wellcome Foundation (now part of GSK) publicized the discovery of fakes of its antibiotic Septrin (co-trimoxazole) that lacked any active ingredients, and the company's efforts to stop its production. Wellcome also had reports that the fakes were being imported into the UK, which it made public along with the warning that it sent to the British Embassy in Bangkok [14]. In 2001, GSK made public the discovery of fakes of its AIDS treatment Combivir (zidovudine + lamivudine) [32], and Genentech publicized information on fakes of Neupogen (filgrastim) [33].

The Pharmaceutical Research and Manufacturers of America announced in April 2003 that, from 1 May 2003, its 60 members would voluntarily report to the FDA “within five working days of determining that there is a reasonable basis to believe their product has been counterfeited” [34]. This is an important local development but it should be mandated by law and become a global standard. Indeed, we have not found one country where drug companies have a legal duty to report discoveries of counterfeits of their products to public health or trade authorities.

## The Sharing of Information on Counterfeit Medicines

We suggest that the pharmaceutical industry, which is such a benefit to our health, is harming both patients and itself by not vigorously warning the public of fake products when they arise. Apart from the moral imperative, there is the prospect of growing legal pressure on drug companies to take responsibility for fakes of their products. In Britain, there are proposals to introduce a charge of “corporate killing” for companies who have contributed to the deaths of customers [35] that could also apply to drug companies if they do not take reasonable steps to warn the public of a fake product.

## Drug Companies Sued in the US

Already, the US has seen the first court case brought against two drug companies for allegedly failing to act to protect customers over a fake drug discovery. In 2002, a Kansas City pharmacist was jailed for diluting the anticancer drugs Gemzar (gemcitabine) and Taxol (paclitaxel). The victims and dead patients' families sued the drug companies, Eli Lilly and Myers Squibb, for not taking steps to stop him. The companies argued that they had no duty to protect the plaintiffs from the pharmacist's criminal acts, but a newspaper reported that Eli Lilly and Myers Squibb settled out of court, apparently for US$72 million, avoiding a legal precedent that would hold drug companies liable for not disseminating such information [36,37].

Chris Jenkins suggests that the PSI could face a legal challenge to open its fake drug databases (E-mail, 9 December 2004): “Only the PSI had an overview of the known racket…In theory, every fake drug case reported by the companies should be on there.” He is concerned that private investigators could be liable for fake drug data they obtain for client companies.

## Governments Must Enforce a Legal Responsibility

We believe that the industry, along with pharmacists, health workers, and governments, needs to extend the “behind the scenes” fight against fakes to a public collaborative approach with a legal responsibility to report suspected counterfeits to drug regulatory authorities, in a similar way to the reporting of “notifiable” infectious diseases. The drug regulatory authorities, accountable to the consumers of drugs, should have a statutory duty to investigate and disseminate the information, with the interests of patients as the prime concern. Drug regulatory authorities in economically poor countries will need additional financial support.

We recognize that false information could seriously damage a company and that information should be verified and used prudently. We also recognize that careful public information measures will be needed to prevent patients from stopping the use of genuine products, but suggest that this is possible as pharmaceutical companies can, and have, alerted the public in collaboration with government agencies (see above). However, the decision to warn the public should not be made by the pharmaceutical industry alone, which has a serious conflict of interest. We believe that the long-term interests of both the industry and patients are best served by more openness and social responsibility to public health. Company staff and shareholders should not be in a position to adjudicate conflicts between commercial gain and public health—such adjudication should be in the hands of government departments accountable to the public.

## Aviation Industry Model

The UK Civil Aviation Authority provides a model: suspected unapproved aircraft parts must, by law, be reported to it [38]. When a report of a counterfeit drug is confirmed, the drug regulatory authorities should be responsible for assessing the public health importance of the information and deciding when and how to alert the country's police, trade, customs authorities, and public, and also the drug regulatory authorities of other countries that may be affected, with the assistance of Interpol as required. If a drug regulatory authority is confident, for example, that the fake drug has been intercepted before it has reached the pharmacies, a public alert may not be necessary. The “confusion” reported in the GSK Halfan syrup case also illustrates the great importance for both companies and government departments to keep a secure paper trail of information so that it is clear what has happened and when.

The pharmaceutical company is also a victim of the counterfeiter and should be supported by governmental authorities if it reports promptly. Individuals who report information on counterfeit drugs should remain anonymous and be protected from the criminal counterfeiting underworld, which may exact retribution. International agreements between companies to avoid taking advantage of competitors' misfortunes, when precipitated by rumors or confirmed reports of fake drugs, may facilitate enhanced cooperation within the pharmaceutical industry.

## International Convention against Counterfeit Drugs

The Madrid meeting in 2004 considered a proposed international framework convention on counterfeit drugs, presented by the WHO, to promote international cooperation and the exchange of information [17]. If enacted this could be a very important contribution to improving drug quality. The effective control of the global epidemic of counterfeit and substandard drugs will not be easy, and will need a multifaceted approach: the provision of effective, available, and inexpensive drugs; the enforcement of drug regulation; more openness by governments as to the scale of the problem; more effective police action against the counterfeiters and those who may be corrupt allies within government and industry; enhanced cooperation between the industry, police, customs, and drug regulators; and enhanced education of patients, drug sellers, and health workers [4,5,20]. We urge the industry and governments to act, through the sharing of crucial public health information, to facilitate the protection of patients and improve the quality of an apparently deteriorating drug supply.

## Counterfeit Drug Conference in Paris

On 15–17 March 2005, the Second Global Forum on Pharmaceutical Anticounterfeiting will convene in Paris, where representatives of the major pharmaceutical companies, governments, medical and scientific professionals, law enforcement agencies, nongovermental organizations, and private investigators will meet to discuss the growing problem that threatens patients and the pharmaceutical industry (Figure 3 [Fig pmed-0020100-g004]).

**Figure:**
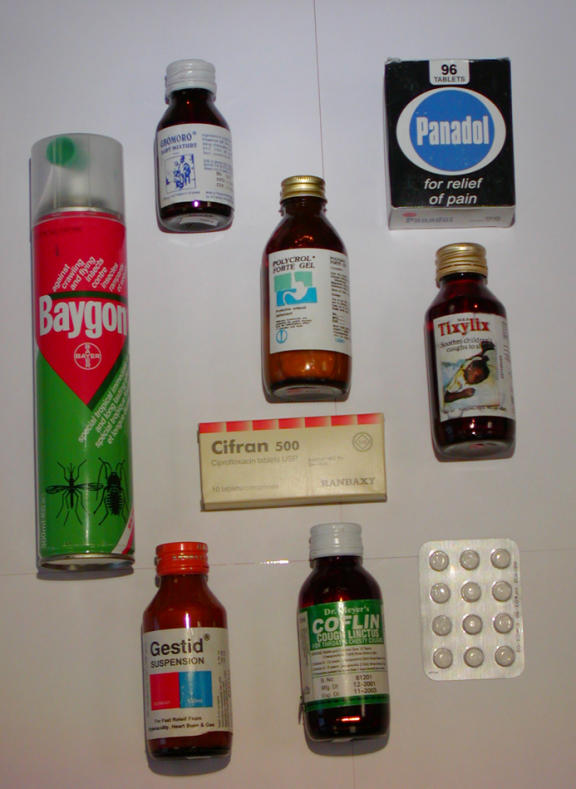
A collection of counterfeit pharmaceutical drugs seized by the NAFDAC in Nigeria (Photograph: NAFDAC/International Chamber of Commerce Counterfeiting Intelligence Bureau)

## Abbreviations

FDA: US Food and Drug Administration
GSK: GlaxoSmithKline
NAFDAC: Nigerian National Agency for Food and Drug Administration and Control
PSI: Pharmaceutical Security Institute
WHO: World Health Organization

## References

[pmed-0020100-b1] Cockburn R (2002). Crime, fear and silence: Making public the fake pharmaceutical drug racket [presentation].

[pmed-0020100-b2] (2003 November 11). Global rise in use of fake drugs.

[pmed-0020100-b3] World Health Organization (1992). Counterfeit drugs—Report of a WHO/IFPMA Workshop, 1992. WHO/DMP/CFD/92.

[pmed-0020100-b4] World Health Organization (1998). Report of the International Workshop on Counterfeit Drugs. WHO/DRS/CFD/98.1.

[pmed-0020100-b5] Newton PN, Rozendaal J, Green M, White NJ (2002). Murder by fake drugs—Time for international action. BMJ.

[pmed-0020100-b6] Saywell T, McManus J (2002 February 21). What's in that pill?. Far East Econ Rev.

[pmed-0020100-b7] Newton PN, Proux S, Green M, Smithuis F, Rozendaal J (2001). Fake artesunate in southeast Asia. Lancet.

[pmed-0020100-b8] Cockburn R (1989 April 18). Warnings delayed over fake asthma inhalers. The Times (London).

[pmed-0020100-b9] (2001 September 4–10). Bad medicine. Tempo (Jakarta).

[pmed-0020100-b10] Crampton T (2003 August 25). Fake malaria pills haunt Asians. International Herald Tribune.

[pmed-0020100-b11] Chakravarty S, Unnithan S, Ram A (2001 January 29). Deadly doses. India Today.

[pmed-0020100-b12] Fackler M (2002 July 29). China's fake drugs kill thousands. San Francisco Examiner.

[pmed-0020100-b13] Cockburn R (1982 December 10). Counterfeit drugs—The other killer in Lebanon. The Guardian (London).

[pmed-0020100-b14] Cockburn R (1984 June 29). Counterfeit drugs that are kill rather than cure. Guardian (London).

[pmed-0020100-b15] World Health Organization (2001). Counterfeit drug reports: 1999—October 2000 [table]. www.who.int/medicines/library/pnewslet/pn300cfd.html.

[pmed-0020100-b16] Rago L (2002). Counterfeit drugs: Threat to public health [presentation].

[pmed-0020100-b17] Gibson L (2004). Drug regulators study global treaty to tackle counterfeit drugs. BMJ.

[pmed-0020100-b18] (2003 October 1).

[pmed-0020100-b19] (1989 November 29). More UK debate on counterfeits. Scrip.

[pmed-0020100-b20] World Health Organization Department of Essential Drugs and Other Medicines (1999). Counterfeit drugs: Guidelines for the development of measures to combat counterfeit drugs.

[pmed-0020100-b21] Moken MC (2003). Fake pharmaceuticals: How they and relevant legislation or lack thereof contribute to consistently high and increasing drug prices. Am J Law Med.

[pmed-0020100-b22] Reidenberg MM, Conner BA (2001). Counterfeit and substandard drugs. Clin Pharmacol Ther.

[pmed-0020100-b23] Csillag C (1998). Epidemic of counterfeit drugs causes concern in Brazil. Lancet.

[pmed-0020100-b24] Broussard P (1996 October 26). Du Nigeria au Niger, d'étranges vaccins contre la méningite. Le Monde.

[pmed-0020100-b25] Hay SI, Guerra CA, Tatem AJ, Noor AM, Snow RW (2004). The global distribution and population at risk of malaria: Past, present, and future. Lancet Infect Dis.

[pmed-0020100-b26] Chryss J (2004). Transcript of “File on 4”: Counterfeit drugs [transcript of radio program]. Program number 04VY3040LHO.

[pmed-0020100-b27] Wolff JC, Thomson LA, Eckers C (2003). Identification of the ‘wrong’ active pharmaceutical ingredient in a counterfeit Halfan drug product using accurate mass electrospray ionisation mass spectrometry, accurate mass tandem mass spectrometry and liquid chromatography/mass spectrometry. Rapid Comm Mass Spectrom.

[pmed-0020100-b28] Nigerian National Agency for Food and Drug Administration and Control (2002 June). List of identified fake products [June 2002]. NAFDAC ref 20-12.

[pmed-0020100-b29] (2003 April 7). GlaxoSmithKline case study. Judicial Protection of IPR in China.

[pmed-0020100-b30] Dunea G (1983). Death over the counter. BMJ.

[pmed-0020100-b31] Schwab D (2003 September 21). Protecting Procrit. Sunday Star-Ledger.

[pmed-0020100-b32] James JS (2001 May 31). Counterfeit drugs: Check Combivir, Serostim, Epogen. AIDS Treat News.

[pmed-0020100-b33] Charatan F (2001). Fake prescription drugs are flooding the United States. BMJ.

[pmed-0020100-b34] US Food and Drug Administration (2003 April 22). FDA commends drug industry commitment to report counterfeit drugs. http://www.fda.gov/bbs/topics/NEWS/2003/NEW00894.html.

[pmed-0020100-b35] (2003). Who will take responsibility for corporate killing?. Lancet.

[pmed-0020100-b36] Morris M (2002 December 6). Pharmacist gets 30 years for diluting medicines. Kansas City Star.

[pmed-0020100-b37] Margolies D (2003 February 9). Payments set for Courtney victims. Kansas City Star.

[pmed-0020100-b38] UK Civil Aviation Authority (2001 October 29). The problem of bogus parts. Airworthiness Notice number 19.

[pmed-0020100-b39] Dondorp AM, Newton PN, Mayxay M, Van Damme W, Smithuis FM (2004). Fake antimalarials in Southeast Asia are a major impediment to malaria control: Multinational cross-sectional survey on the prevalence of fake antimalarials. Trop Med Int Health.

[pmed-0020100-b40] Yardley J (2004 May 5). Infants in Chinese city starve on protein-short formula. New York Times.

[pmed-0020100-b41] Rudolf PMM, Bernstein IBG (2004). Counterfeit drugs. N Engl J Med.

[pmed-0020100-b42] Raufu A (2003). India agrees to help Nigeria tackle the import of fake drugs. BMJ.

[pmed-0020100-b43] (1995). Fake drugs: A scourge of the system. WHO Drug Inf.

[pmed-0020100-b44] Hanif M, Mobarak MR, Ronan A, Rahman D, Donovan J (1995). Fatal renal failure caused by diethylene glycol in paracetamol exlixir: The Bangladesh epidemic. BMJ.

[pmed-0020100-b45] O'Brien KL, Selanikio JD, Hecdivert C, Placide MF, Louis M (1998). Epidemic of pediatric deaths from acute renal failure caused by diethylene glycol poisoning. JAMA.

[pmed-0020100-b46] Ahmad K (2004). Antidepressants are sold as antiretrovirals in DR Congo. Lancet.

[pmed-0020100-b47] (2005 January). Warning: At least seven types of fake artesunate are being sold in mainland SE Asia. Wellcome Trust SE Asian Tropical Medicine Research Units.

