# Correction: The Global Threat of Counterfeit Drugs: Why Industry and Governments Must Communicate the Dangers

**DOI:** 10.1371/journal.pmed.0040289

**Published:** 2007-09-25

**Authors:** Robert Cockburn, Paul N Newton, E. Kyeremateng Agyarko, Dora Akunyili, Nicholas J White

Correction for:

Cockburn R, Newton PN, Agyarko EK, Akunyili D, White NJ (2005) The Global Threat of Counterfeit Drugs: Why Industry and Governments Must Communicate the Dangers. PLoS Med 2(4): e100. doi:10.1371/journal.pmed.0020100

The authors quoted a news story from the *San Francisco Examiner* [12] that states that 192,000 people died in China in 2001 as a consequence of fake drugs. This story was sourced to the *Shenzhen Evening News* of January 25, 2002. However, the authors are grateful to Professor Jin Shaohong, Executive Director-General of the National Institute for the Control of Pharmaceutical and Biological Products in Beijing, China, for pointing out that the English translation of the *Shenzhen Evening News* story used by the *San Francisco Examiner* and other newspapers is incorrect. The article does not mention counterfeit drugs, but claims that 192,000 people died of drug-induced diseases from the irrational use of drugs in 2001. As this statistic is often quoted in the literature on counterfeit pharmaceuticals, the authors wish to correct this error.

Further discussion by three of the authors of this correction is available at http://medicine.plosjournals.org/perlserv/?request=read-response&doi=10.1371/journal.pmed.0020100#r1765.

